# Everolimus Implicated in Case of Severe Gastrointestinal Hemorrhage

**DOI:** 10.1155/2017/3657812

**Published:** 2017-06-28

**Authors:** Paul Gonzales, Seth Klusewitz, Johanna Marowske, John Gancayco, Michael B. Osswald, Robert Setlik

**Affiliations:** ^1^Department of Internal Medicine, San Antonio Military Medical Center, Fort Sam Houston, San Antonio, TX 78234, USA; ^2^Gastroenterology Service, San Antonio Military Medical Center, Fort Sam Houston, San Antonio, TX 78234, USA; ^3^Hematology/Oncology Service, San Antonio Military Medical Center, Fort Sam Houston, San Antonio, TX 78234, USA

## Abstract

Breast cancer remains the leading cause of cancer and the third leading cause of cancer related deaths among our population with an estimated number of 246,660 new cases and 40,450 deaths in 2016. With treatment advancements, including targeted agents such as Everolimus, a mammalian target of rapamycin (mTOR) inhibitor, survivability and quality of life continue to improve. However, with the use of these agents come adverse effects, some of which are still being characterized. Our case demonstrates recurrent episodes of gastrointestinal bleeding in a 60-year-old woman being treated with Everolimus for progressive metastatic breast cancer. On endoscopy, bleeding was secondary to erosive gastritis. Previous case reports have described bleeding due to gastric antral vascular ectasia (GAVE), which was described in two prior reported cases. In our case, bleeding also occurred on a reduced dose of Everolimus compared to what is previously reported (5 mg versus 10 mg). As a result of her gastrointestinal bleeding, she required multiple endoscopic interventions including argon plasma coagulation and multipolar heater probe to achieve hemostasis. This is the first case reported of gastrointestinal bleeding not consistent with GAVE and occurring while being on a reduced dose of Everolimus. It is important to document our case so that the Gastroenterology and Hematology communities can be educated and made aware for their patient populations on Everolimus.

## 1. Introduction

Targeted agents, such as the mTOR inhibitor Everolimus, have increased the progression-free survival and quality of life of patients with numerous types of cancer. These targeted agents, while typically well-tolerated, have unique side effect profiles many of which are still being characterized. Given that patients treated with these agents are surviving longer, knowledge and mitigation of their side-effects are of paramount importance.

Since July 2012, the US Food and Drug Administration (FDA) has approved the use of Everolimus in combination with Exemestane for use in postmenopausal women with advanced hormone-receptor positive, HER2-negative breast cancer after failure of treatment with Letrozole or Anastrozole [1]. The FDA prescribing information addresses adverse reactions in a number of organ systems. Specifically, it lists the most common treatment-emergent, gastrointestinal adverse reactions as being stomatitis, diarrhea, abdominal pain, nausea, vomiting, constipation, and dry mouth. Notably, hemorrhage from all causes was only described in 3% of patients without specific mention of gastrointestinal bleeding [1].

Herein, we describe a patient with multiple episodes of life-threatening gastrointestinal bleeding while undergoing therapy with Everolimus with resolution following removal of therapy. As described by Fujihara et al. [2] and Assi and Abdel-Samad [3], there have been only 2 other reports of mTOR inhibitors and only 1 report of Everolimus implicated in life-threatening gastrointestinal bleeding. The case report describing Everolimus described gastrointestinal bleeding secondary to gastric antral vascular ectasia (GAVE); however we present a unique case in a patient with life-threatening gastrointestinal bleeding not consistent with GAVE. During the review of literature, this is the only case not consistent with GAVE and on reduced dose Everolimus and, given the potential life-threatening implications of this adverse reaction, awareness and vigilance are critical for any physician using this agent.

## 2. Case Description

**Figure 1:**
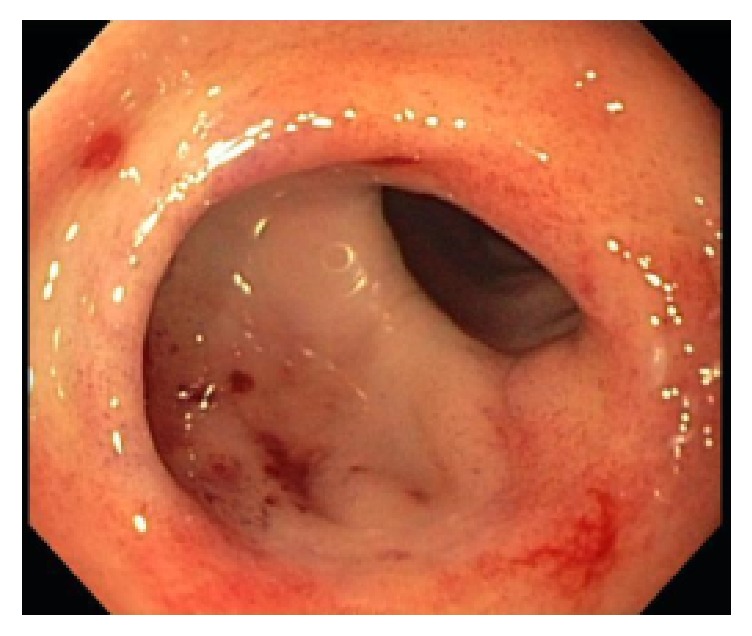
Antral stomach, several small punctate lesions observed.

Our patient is a 65-year-old Caucasian female with past medical history of estrogen receptor (ER) positive, progesterone receptor (PgR) negative, HER2 negative metastatic breast cancer treated with Everolimus and Fulvestrant after disease progression on first-line Anastrozole [4]. She initially started Fulvestrant as a single agent per the CONFIRM trial and then started Everolimus 5 mg daily one month later [5]. This treatment plan was devised in light of the patient's concerns over treatment-related side effects. Her initial gastrointestinal bleed began one month after starting Everolimus. On presentation, she had complaints of weakness, fatigue, and endorsed several episodes of melena. She was found to be anemic with hemoglobin of 6.1 g/dL, down from 11.7 g/dL (reference range 11.0–16.0 g/dL) just one week priorly. She was admitted for anemia secondary to suspected upper gastrointestinal bleed and after admission had further decrease of hemoglobin to 5.0 g/dL. She was transfused two units of packed red blood cells (pRBCs) with an appropriate rise in hemoglobin to 8.4 g/dL. Upper endoscopy performed by Gastroenterology revealed erosive gastritis. Multiple flat erosions with blood oozing were identified within the antral stomach (Figure 1). Hemostasis of these lesions was achieved with injections of epinephrine into the surrounding mucosa. One day following upper endoscopy, she required an additional transfusion of one unit of packed red blood cells for hemoglobin of 6.9 g/dL, again with appropriate response. She had no further melenic stools during her hospitalization and her hemoglobin stabilized at 9.2 g/dL. She was given prescription for Pantoprazole 20 mg twice daily and discharged home. Everolimus was held for two weeks, while continuing therapy with Fulvestrant.

**Figure 2:**
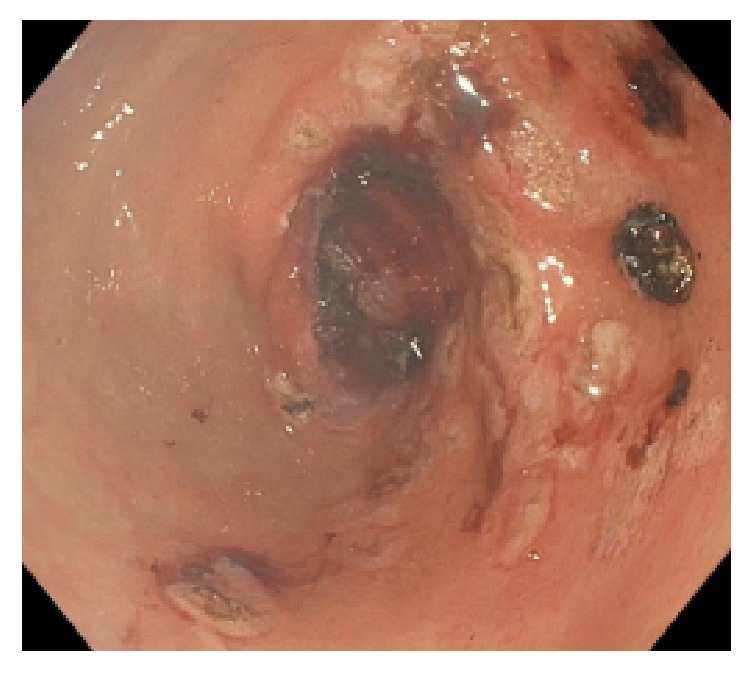
Following intraprocedural ERBE circumferential probe argon plasma coagulation, hemostasis was achieved.

Three weeks following discharge, the patient again developed index symptoms of fatigue, weakness, and melena after restarting Everolimus seven days priorly. The patient was admitted to an outside institution and transfused 8 units of pRBCs over the course of a two-week admission. During this time, she underwent both upper and lower endoscopy with upper endoscopy demonstrating friable mucosa, with no location of frank blood loss being identified. Lower endoscopy revealed normal mucosa with several nonbleeding diverticula and additional testing with radiologic mesenteric angiography was negative at that time. She was subsequently transferred to our hospital and hemoglobin on arrival was noted to be 6.5 g/dL. She was transfused two units of pRBCs with follow-up blood counts demonstrating adequate hemoglobin response to 8.9 g/dL. Gastroenterology was reconsulted and performed repeat upper endoscopy which demonstrated diffuse erosions with active oozing of blood in the gastric antrum. Hemostasis was achieved via intraprocedural ERBE circumferential probe argon plasma coagulation (Figure 2). Following her endoscopy, her hemoglobin level stabilized and melenic stools resolved. She was discharged home with hemoglobin of 9.2 g/dL, provided a prescription for Sucralfate 1 g twice daily, and increased dosing of her Pantoprazole to 40 mg twice daily. Everolimus treatment was permanently discontinued upon discharge.

**Figure 3:**
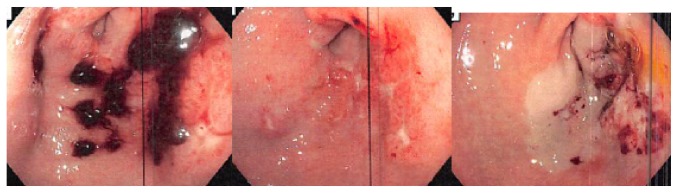
BICAP multipolar heater probe and epinephrine application following additional endoscopy.

Two weeks afterward, she was again admitted for symptomatic anemia with hemoglobin of 7.7 g/dL. Upper endoscopy was repeated which illustrated moderate clotted blood in the area of the gastric antrum with multiple 2-3 mm stellate, clean-based ulcers discovered following washing in the setting of friable mucosa. Definitive therapy was pursued with BICAP multipolar heater probe and epinephrine (Figure 3). The patient was subsequently discharged with stable hemoglobin and was seen in Hematology/Oncology Clinic one week later. Despite rising Cancer Ag 27–29 and increased tumor burden evidenced by CT-scan, it was decided that Everolimus therapy would not be restarted in light of the risk of continued gastrointestinal bleeding. The patient was started on palliative Capecitabine and has continued to have stable hemoglobin values since that time with no reoccurrences of gastrointestinal bleeding. She has not required further endoscopic evaluation since discharge.

## 3. Discussion

In two previous case reports, antineoplastic chemotherapy with an mTOR-based regimen was associated with gastric bleeding consistent with gastric antral vascular ectasia (GAVE). Despite our patient receiving a 50% Everolimus dose-reduction, she also developed gastric bleeding. However, our patient's endoscopic appearance is not consistent with typical findings in GAVE. In patients with GAVE, diagnosis is established with biopsy and visual evaluation on endoscopy demonstrating bleeding with appearance of red stripes radiating from the antrum of the stomach to the pylorus commonly described as watermelon stomach [6]. Our case is unique in that our patient had diffuse erosive gastritis with ulcerations likely secondary to the mTOR inhibitors which has not been demonstrated in the literature previously.

In our case, the patient required multiple modalities of endoscopic interventions to achieve hemostasis, ranging from mucosal injections of epinephrine to plasma argon coagulation and multipolar heater probes. While hemorrhage was adequately controlled in her case using more conventional methods, it is vital to have an understanding of all possible therapies available when treating life threatening hemorrhages, particularly associated with malignancy and their directed therapies. Of interest, the Ankahert blood stopper is a standardized mixture of plants, which acts as a topical hemostatic agent acting on the endothelium, blood cells, angiogenesis, cellular mediators and cellular proliferation and is becoming an effective alternative to conventional hemostatic measures [7]. In a literature review performed by Beyazit et al. [7], the Ankahert blood stopper was successfully used in the management of gastrointestinal hemorrhages from multiple etiologies including malignant and radiation associated bleeding. Additionally, in a case report by Ozdemir et al. [8], the blood stopper was successfully used in the management of bevacizumab-associated gastrointestinal hemorrhage.

In both prior instances of gastrointestinal bleeding associated with mTOR inhibitors described in case reports by Fujihara et al. [2] and Assi and Abdel-Samad [3], GAVE was identified as the likely etiology of bleeding. In our case, the patient's gastrointestinal bleeding was not only temporally associated with initiation of therapy but also resolved following its termination implicating Everolimus as the causative agent. As targeted drug therapy becomes more widely used in not only breast but many forms of cancer, knowledge of potentially life threatening adverse effects is of great importance to empower the caring physician to discontinue therapy when these events are recognized. This case further contributes to the literature that substantiates life-threatening gastrointestinal bleeding as a potential serious adverse outcome to Everolimus therapy.
